# RNF5 inhibits HBV replication by mediating caspase-3-dependent degradation of core protein

**DOI:** 10.3389/fmicb.2025.1548061

**Published:** 2025-04-01

**Authors:** Jing Xu, Hongxiao Song, Fengchao Xu, Yanli Gao, Hongyu Jiang, Guangyun Tan

**Affiliations:** ^1^Department of Hepatology, Center for Pathogen Biology and Infectious Diseases, Institute of Translational Medicine, The First Hospital of Jilin University, Changchun, Jilin, China; ^2^Health Examination Center, The First Hospital of Jilin University, Changchun, Jilin, China; ^3^Department of Pediatrics, The First Hospital, Jilin University, Changchun, Jilin, China

**Keywords:** RNF5, HBV - hepatitis B virus, core protein, Caspase-3, E3 ubiqitin ligase

## Abstract

The RING finger protein 5 (RNF5), an E3 ubiquitin ligase, has demonstrated significant antiviral activity against various viruses, including severe acute respiratory syndrome coronavirus 2 (SARS-CoV-2) and Kaposi’s sarcoma-associated herpesvirus (KSHV). However, its role in hepatitis B virus (HBV) replication has not been previously studied. In this study, we demonstrate that RNF5 effectively inhibits HBV replication by promoting the degradation of the HBV Core protein through a Caspase-3-dependent pathway. We first show that RNF5 expression is upregulated in HBV-infected cells and patient samples, suggesting a role in the host’s antiviral response. Subsequently, we investigate the mechanism by which RNF5 mediates its antiviral effect, finding that RNF5 targets the Core protein for degradation independently of its E3 ubiquitin ligase activity. The degradation of Core protein is mediated through a Caspase-3-dependent mechanism rather than the proteasomal pathway. Interestingly, RNF5’s antiviral function does not rely on ubiquitination, indicating an alternative pathway involving apoptosis-related processes. These findings highlight the multifunctional role of RNF5 and suggest that targeting RNF5 could serve as a novel therapeutic approach to control HBV replication, providing new insights into the development of antiviral therapies against HBV.

## Graphical Abstract

RNF5 is induced in HBV-replicating cells and inhibits HBV by recognizing Core protein via protein-protein interaction, and Caspase-3 is essential for RNF5-mediated Core degradation. 
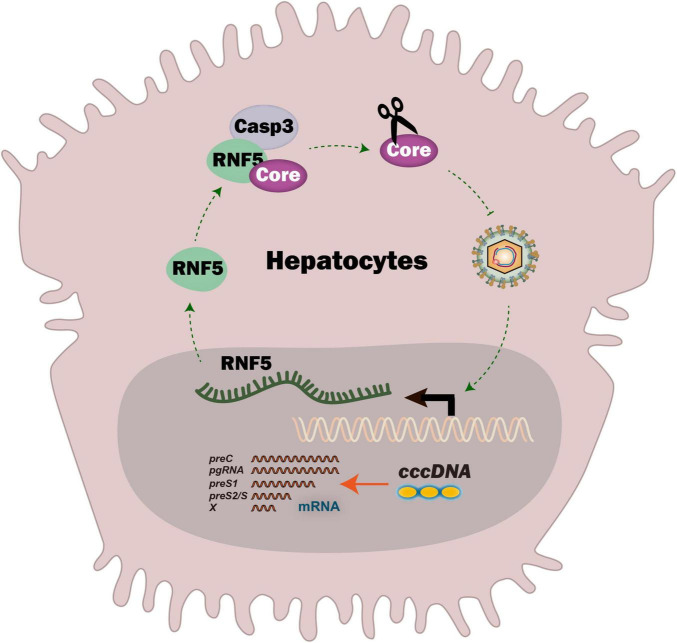

## 1 Introduction

Hepatitis B virus (HBV) infection remains a major global health challenge, affecting more than 250 million people worldwide and causing significant morbidity and mortality due to its association with chronic hepatitis, cirrhosis, and hepatocellular carcinoma (HCC) (Hui et al., 2024; Jeng et al., 2023; Tan et al., 2018a). Despite the availability of an effective vaccine and antiviral therapies that suppress viral replication, a complete cure remains elusive. The persistence of covalently closed circular DNA (cccDNA) in the nucleus of infected hepatocytes is the main barrier to achieving a functional cure (Levrero et al., 2009; Nassal, 2015).

During its lifecycle, HBV genome produces five RNA transcripts (3.5 kb, 2.4 kb, 2.1 kb, and 0.7 kb), encoding seven proteins essential for replication and infection. These include the core protein (HBc), forming the nucleocapsid, and the pre-core protein, processed into hepatitis B virus e antigen (HBeAg) to modulate immune responses. The surface proteins (large, middle, and small) form the viral envelope, while the polymerase (Pol) drives genome replication. The hepatitis B virus X protein (HBx) regulates viral replication and host pathways, contributing to persistence and potential oncogenesis (Song et al., 2021; Tan et al., 2018b; Tan et al., 2019; Wang et al., 2023; Wei et al., 2022; Xu et al., 2019; Xu et al., 2022), highlighting the coordinated functions of these viral components. HBV Core protein plays an essential role in multiple stages of the HBV life cycle, including pre-genomic RNA (pgRNA) encapsulation, capsid assembly, and interaction with host factors to evade immune responses (Ghany et al., 2021; Mak et al., 2017; Nair and Zlotnick, 2021; Xu et al., 2024). Therefore, targeting the HBV Core protein has emerged as a promising strategy for HBV treatment (McFadden and Sarafianos, 2023). Capsid assembly modulators (CAMs), which target the HBV Core protein and disrupt capsid assembly, have shown promise in clinical trials as potential therapeutic agents for chronic hepatitis B (CHB) (McFadden and Sarafianos, 2023; Popping et al., 2019; Taverniti et al., 2024). CAMs can misdirect capsid assembly, leading to non-functional capsids and reduced HBV replication. However, these compounds are still under investigation, and there is a need for additional therapeutic targets that can complement or enhance the efficacy of existing treatments.

The ubiquitin-proteasome system (UPS) is a key pathway for protein degradation and regulation of cellular homeostasis (Atani et al., 2024). The UPS plays a pivotal role in the regulation of viral protein stability, which in turn impacts viral replication. In the case of HBV, understanding how the UPS interacts with viral proteins, particularly the core protein, could lead to new therapeutic strategies that target these pathways to prevent or inhibit HBV replication. Additionally, recent research suggests that host E3 ligases may regulate viral protein degradation through non-canonical, ubiquitin-independent mechanisms, further expanding the therapeutic potential of the UPS in HBV treatment (Kar et al., 2024; Kong et al., 2019).

The RING finger protein 5 (RNF5) is an E3 ubiquitin ligase that has been implicated in the degradation of viral and cellular proteins, thus playing a role in regulating various cellular processes, including immune responses and protein homeostasis (Didier et al., 2003; Ge and Zhang, 2023; Liu and Xia, 2022; Yan et al., 2023; Zhong et al., 2009). Previous studies have demonstrated the antiviral activity of RNF5 against a range of viruses, including severe acute respiratory syndrome coronavirus 2 (SARS-CoV-2) and Kaposi’s sarcoma-associated herpesvirus (KSHV), through the ubiquitination and degradation of viral proteins (Li Z. et al., 2023; Li X. et al., 2023). However, the role of RNF5 in the context of HBV infection has not been explored. In this study, we found that the E3 ubiquitin ligase RNF5 inhibits HBV replication by promoting the degradation of the HBV core protein through a Caspase-3-dependent pathway. Notably, the antiviral effect of RNF5 is independent of its ubiquitin ligase activity, suggesting that it regulates viral protein stability through a non-canonical mechanism. Our findings provide new insights into the role of RNF5 in HBV infection and lay the groundwork for the development of therapeutic strategies targeting the HBV core protein.

## 2 Materials and methods

### 2.1 Sample collection

This study enrolled 44 HBV-infected patients and 19 healthy controls (Supplementary Tables 1–3). Venous blood samples (5 mL) were collected to extract serum and peripheral blood mononuclear cells (PBMC). HBV DNA levels were measured using Roche’s COBAS TaqMan Kit, and liver function along with biochemical parameters was assessed using an automatic biochemical analyzer. These procedures were conducted in the Department of Hepatology, First Hospital of Jilin University, Changchun, China.

### 2.2 Cell lines and antibodies

HEK293T, HepG2, HepAD38, HepG2.2.15 and HepG2-NTCP cell lines were cultured in dulbecco’s modified eagle medium (DMEM) supplemented with 10% heat-inactivated fetal bovine serum (FBS), 100 IU/mL penicillin, and 100 mg/mL streptomycin at 37°C in a 5% CO2 incubator. The following primary antibodies were used: HA (Proteintech, 51064-2-AP), GAPDH (Proteintech, 10494-1-AP), Flag (Sigma, F3165), Tubulin (Calbiochem, cp06), Actin (Wanleibio, WL01372), RNF5 (Santa Cruz Biotechnology, 22B3), GST (Cell Signaling, 2622) and Caspase-3 (Cell Signaling, 9662S), PSMD2 (Proteintech, 14748-1-AP), the Core antibody was generously provided by Professor Bin Ju. All antibodies were diluted to 1:1,000 with antibody diluent (absin, abs9299).

### 2.3 Infusion cloning

The Core expression construct was generated by cloning the coding sequence of the target human gene into the VR1012 vector tagged with Flag, HA, or GST using the EASY-Uni Seamless Cloning Kit (TransGen, Beijing, China). Site-directed mutagenesis of Core was performed using the QuikChange PCR method (TransGen). The primers used are detailed in Supplementary Table 4.

### 2.4 RNA and DNA extraction and quantitative real-time PCR

Total RNA was extracted with TRIzol (Invitrogen, 15596026CN) and reverse-transcribed into cDNA using Superscript III Transcriptase (Invitrogen, 18080093). HBV DNA was extracted from cell lysates or culture supernatants following the manufacturer’s instructions (TransGen, Beijing, China). cDNA was obtained by reverse transcription with 1 μg RNA and then diluted 10-fold to be used as template. qRT-PCR was performed using gene-specific primers (Supplementary Table 4), with GAPDH as an internal control. The qRT-PCR assay was carried out in a 20 μL volume consisting of 1 μL of 10 μmol/L primers, 5 μL of diluted cDNA templates, 4 μL Nuclease-free water, and 10 μL SYBR Green qPCR Master Mix (Roche, 4913914001). Amplifications of the target fragment were carried out as the following steps: initial activation of the HotMaster Taq DNA Polymerase at 95°C for 10 min, and then followed with 40 cycles of 95°C for 10 s and 60°C for 30 s.

### 2.5 Co-immunoprecipitation and western blotting

Cells were seeded in 6-well plates (HEK293T 7.0 × 10^5^ cells, HepG2 5.5 × 10^5^ cells) or 12-well plates (HEK293T 2.5 × 10^5^ cells, HepG2 2.0 × 10^5^ cells) overnight, transfected by Lipofectamine™ 3000 Reagent (Invitrogen, L3000015) according to the manufacturer’s instructions, and cells were harvested at 24–72 h post-transfection. Cell lysates were prepared using lysis buffer (50 mM Tris-HCl, pH 8.0, 150 mM NaCl, 1% NP-40) containing protease inhibitors (Roche, United States). For Co-IP, lysates were incubated overnight with ANTI-FLAG^®^ M2 or ANTI-HA^®^ M2 Affinity Gel (Sigma, United States). For Western blotting, proteins were quantified using the Coomassie Plus™ Protein Assay reagent (Thermo Scientific, Rockford, IL, United States). Immunoblotting followed standard protocols, using SDS-PAGE separate proteins, PVDF membrane transfer, blocking in Phosphate Buffered Saline (PBS) with 0.1% Tween-20 and 5% Bovine Serum Albumin (BSA), and incubation with appropriate antibodies. Chemi-luminescence was measured with ECL (Millipore) and band intensities were quantified using ChemiDoc™ XRS + software (Bio-Rad).

### 2.6 Immunofluorescence

HepG2 cells (0.8 × 10^5^) were transfected with HA-Core, Flag-RNF5-WT, or RNF5-C42S plasmids for 48 h, then fixed in 1:1 acetone-methanol at 37°C for 10 min. After blocking with 5% BSA in PBS-Triton, cells were incubated with primary antibodies Flag (mouse) and HA (rabbit), followed by fluorescently labeled secondary antibodies goat anti-mouse IgG conjugated with fluorescein isothiocyanate (FITC)- or both Cy3 (rabbit)- and FITC (mouse)-conjugated IgG (Proteintech). Nuclei were stained with 4’,6-diamidino-2-phenylindole (DAPI) (proteintech). Fluorescence images were acquired using a fluorescence microscope.

### 2.7 ELISA

Supernatants from HepG2 cells transfected with RNF5-WT, RNF5-C42S, or EV and pHBV1.2 plasmids were collected 24, 48, or 72 h post-transfection. HBeAg and HBsAg levels were measured using an ELISA kit (Kehua Shengwu, China).

### 2.8 CRISPR/Cas9 knockout

HepG2, HepAD38 or HepG2-NTCP cells (1.0 × 10^5^) were seeded in 24-well plates overnight, transfected with Cas9 and Caspase-1, -3, -4, and -8 sgRNA (FG-EH-Cas9-2F-PPW) along with a puromycin-resistant gene plasmid (PL-GFP-IP) using ViaFect (Promega). At 36 h post-transfection, cells were selected with puromycin (2 μg/mL) or subjected to immunoblotting. Single-cell clones were expanded in 96-well plates and validated via DNA sequencing and immunoblotting. sgRNA sequences are provided in Supplementary Table 4.

### 2.9 Virus production and cell infection

HBV particles were collected and concentrated from the supernatant of HepAD38 cells. Cell supernatants were collected, and according to the process of PEG-it Virus Precipitation Solution (SBI, United States) to collect HBV particles. During infection, HepG2-NTCP cells (1.0 × 10^5^) were seeded in 24-well plates overnight, and HBV particles were diluted in DMEM supplemented with 5% FBS, 2.5% dimethyl sulfoxide (DMSO), 4% PEG8000, 1% penicillin and 1% streptomycin. Infection was performed at a multiplicity of infection (MOI) of 1,000. A 24 h post-infection, the cells were washed 5 times with PBS, and cells were maintained in DMEM containing 2.5% DMSO and 5% FBS, with medium refreshed every 48 h. At each interval, supernatants were collected, clarified by centrifugation (500 × g, 5 min), and cryopreserved at −80°C. On day 7 post-infection, cells were systematically harvested for further experiments.

### 2.10 Statistical analysis

All experiments were performed in triplicate. Data were analyzed using GraphPad Prism 10 and presented as Mean ± SD (measurement data) or case percentages (count data). Two-group differences were evaluated using a two-tailed unpaired *t*-test, and linear regression analysis was used for correlations. Statistical significance was set at *p* < 0.05.

## 3 Results

### 3.1 RNF5 expression is altered in HBV infection and correlates with host response

**FIGURE 1:**
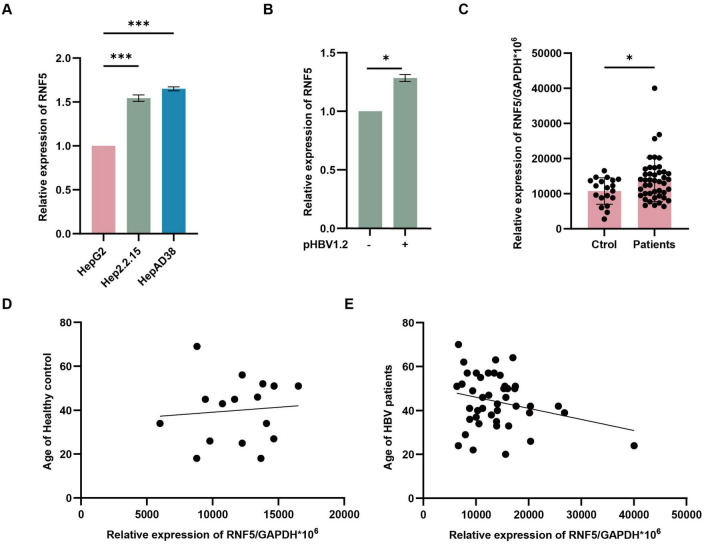
RNF5 expression is altered in HBV infection and correlates with host response. **(A,B)** RNF5 mRNA levels are upregulated *in vitro*. HepG2, Hep2.2.15, and HepAD38 cells were collected, and RNA was extracted for qPCR analysis of RNF5 expression **(A)**. HepG2 cells were transfected with pHBV1.2 plasmids, and RNF5 mRNA levels were evaluated by qPCR at 0, 24, 48, and 72 h **(B)**. **(C–E)** RNF5 mRNA levels are elevated in HBV patients and show age-related differences compared to healthy controls. **(C)** RNF5 mRNA levels were measured by qPCR in PBMCs from healthy controls (*n* = 19) and HBV patients (*n* = 44). **(D,E)** Correlation analysis of RNF5 mRNA levels with age in HBV patients and healthy controls. Data were obtained from patients at the First Hospital of Jilin University. Results are presented as mean ± SD from three independent experiments. *p < 0.05; ***p < 0.001.

To explore the molecular mechanisms underlying virus-host interactions and to understand how host factors influence hepatitis B virus (HBV) replication and persistence, we focused on RNF5, a host factor previously implicated in regulating host-virus interactions (Zhong et al., 2009). RNF5 was significantly upregulated in HepAD38 and HepG2.2.15 cells, both of which support persistent HBV replication, highlighting its potential role in modulating the viral lifecycle (Figure 1A). Similarly, *in vitro* studies showed that RNF5 mRNA levels were induced after pHBV transfection, further suggesting its involvement in the host response to HBV replication (Figure 1B). Interestingly, analysis of clinical samples revealed that RNF5 expression was significantly elevated in HBV patients compared to healthy controls (Figure 1C). Notably, we observed an age-related correlation in RNF5 expression: in HBV patients, higher age was associated with lower RNF5 expression, whereas in healthy individuals, older age corresponded to higher RNF5 expression (Figures 1D,E). This reversal of the typical age-related expression pattern of RNF5 in HBV patients suggests that HBV infection may manipulate RNF5 expression to benefit viral replication during the host-virus interaction process. Collectively, these findings suggest that RNF5 is actively involved in the host response to HBV infection and may play a role in modulating viral replication.

### 3.2 RNF5 inhibits HBV infection and replication

**FIGURE 2:**
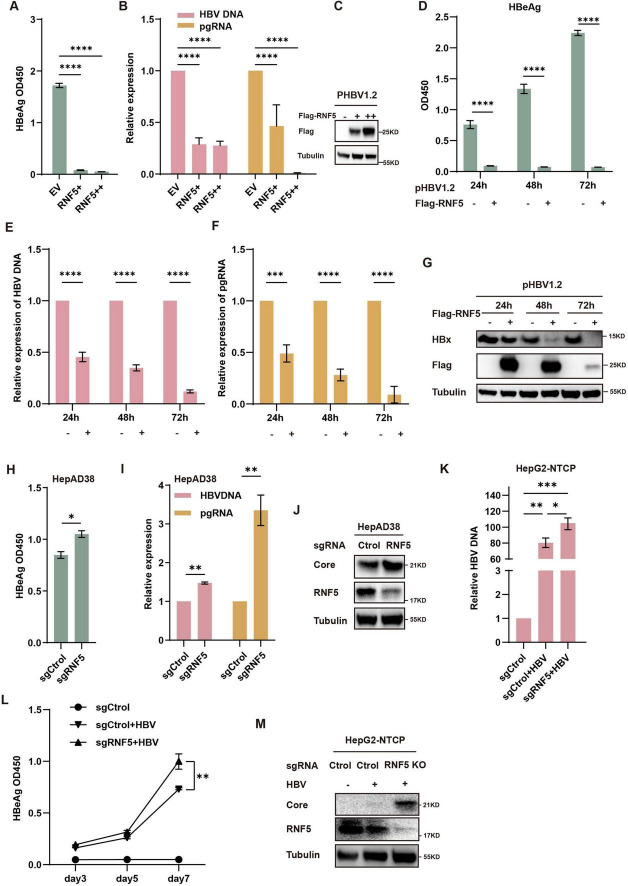
RNF5 inhibits HBV replication. **(A–J)** RNF5 suppresses HBV replication. **(A–C)** HepG2 cells were co-transfected with pHBV1.2 and increasing amounts of Flag-RNF5 or an empty vector (EV). After 48 h, supernatants were analyzed for HBeAg levels via ELISA **(A)**, RNA was extracted for qPCR to measure HBV DNA and pgRNA levels **(B)**, and cell lysates were subjected to immunoblotting with the indicated antibodies **(C)**. **(D–G)** Time-course analysis of RNF5’s effect on HBV replication. HepG2 cells were co-transfected with pHBV1.2 and Flag-RNF5 or EV. Samples were collected at 24, 48, and 72 h for analysis of HBeAg levels **(D)**, HBV DNA and pgRNA levels **(E,F)**, and protein expression by immunoblotting **(G)**. **(H–J)** RNF5 suppresses HBV replication in HepAD38 cells. WT and RNF5 KO HepAD38 cells were seeded in 12-well plates, and after 48 h, samples were collected to analyze HBeAg levels **(H)**, HBV DNA and pgRNA levels **(I)**, and protein expression via immunoblotting **(J)**. **(K–M)** RNF5 suppresses HBV infection. WT and RNF5 KO HepG2-NTCP cells were infected with HBV for 24 h. Supernatants were collected every 2 days, and the medium was changed after washing the cells five times with PBS. Cells were harvested on day 7 post-infection. Samples were analyzed for HBV DNA levels via qPCR **(K)**, HBeAg levels via ELISA **(L)**, and protein expression via immunoblotting **(M)**. *p < 0.05; **p < 0.01; ***p < 0.001; ****p < 0.0001.

To investigate the role of RNF5 in HBV replication, we first tested its impact in HepG2 cells. These cells were co-transfected with varying amounts of RNF5 plasmid alongside HBV-expressing plasmids to evaluate how different levels of RNF5 influence HBV replication. We measured several HBV markers, including HBV DNA, pre-genomic RNA (pgRNA), and HBeAg. Our results demonstrated a clear dose-dependent inhibition of these viral markers (Figures 2A-C), suggesting that RNF5 acts as a potent suppressor of HBV replication. The effects of RNF5 on HBV replication were observed at multiple time points following transfection: 24, 48, and 72 h. At each of these time points, the expression levels of HBV DNA, pgRNA, and HBeAg were consistently reduced (Figures 2D-G). This pattern of inhibition reinforces the hypothesis that RNF5 exerts a strong and persistent antiviral effect against HBV replication, which is sustained over time. To further validate our findings, we investigated the inhibitory effect of RNF5 on HBV replication in HepAD38 cells. We observed that knocking out RNF5 (KO pool) led to elevated levels of HBeAg, HBV DNA, pgRNA, and HBc (Figures 2H-J). To strengthen our conclusions, we conducted HBV infection experiments using WT and RNF5 KO HepG2-NTCP cells (pool). The results showed that following RNF5 knockout, HBeAg levels progressively increased at 3, 5, and 7 days post-infection, while HBV DNA and HBc were significantly upregulated, with a more pronounced increase compared to WT cells (Figures 2K-M). Taken together, these data strongly suggest that RNF5 inhibits multiple stages of the HBV life cycle, including viral replication, transcription, and protein production.

### 3.3 RNF5 inhibits HBV replication independent of its E3 enzyme activity

**FIGURE 3:**
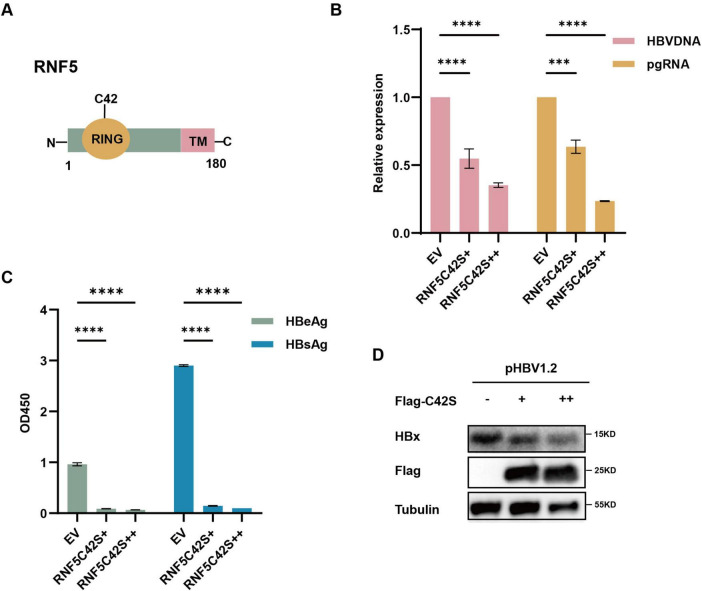
RNF5 inhibits HBV replication independent of its E3 enzyme activity. **(A)** Schematic representation of RNF5 wild-type (WT) and RNF5-C42S mutant structures. **(B–D)** Effect of RNF5 E3 ligase activity on HBV replication. HepG2 cells were co-transfected with pHBV1.2 and increasing amounts of Flag-RNF5-C42S or EV. After 48 h, HBV DNA and pgRNA levels were measured by qPCR **(B)**, HBeAg and HBsAg levels were analyzed by ELISA **(C)**, and HBx protein level was assessed by immunoblotting **(D)**. ***p < 0.001; ****p < 0.0001.

To investigate whether the E3 ubiquitin ligase activity of RNF5 is essential for its antiviral effect, we employed a mutant RNF5 (C42S), which lacks the ability to perform ubiquitination due to the mutation of the catalytic cysteine residue at position 42 (Figure 3A). This RNF5 mutant is unable to catalyze the attachment of ubiquitin molecules to target proteins, which typically leads to their degradation. Surprisingly, RNF5 C42S exhibited similar inhibitory effects on HBV replication as the wild-type RNF5. Despite the absence of E3 ligase activity, the RNF5 C42S mutant still significantly reduced the levels of HBV markers such as HBV DNA, pgRNA, HBeAg, and HBsAg (Figures 3B,C), as well as the HBx protein levels (Figure 3D). These findings suggest that the antiviral action of RNF5 does not solely depend on its E3 ligase activity. Instead, RNF5 appears to inhibit HBV replication through a mechanism independent of its ubiquitin ligase function.

### 3.4 RNF5 mediates degradation of HBV core protein independent of the proteasome pathway

**FIGURE 4:**
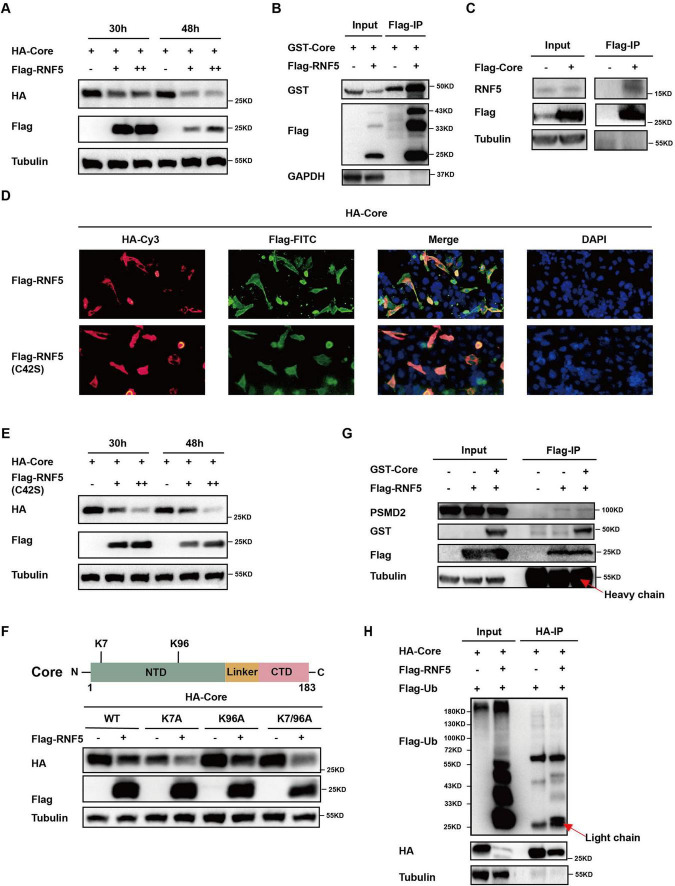
RNF5 mediates degradation of HBV core protein independent of the proteasome pathway. **(A)** Time-course analysis of RNF5-mediated degradation of Core. HepG2 cells were co-transfected with HA-Core and Flag-RNF5-WT or EV. Cell lysates were analyzed by Western blotting at 30 and 48 h post-transfection. **(B,C)** Interaction between RNF5 and Core. Co-IP confirmed interactions between RNF5 and Core **(B)** and between Core and endogenous RNF5 **(C)** in HepG2 cells 28 h post-transfection. **(D)** RNF5 colocalizes with Core protein. HepG2 cells co-transfected with HA-Core and Flag-RNF5-WT, C42S, or EV were subjected to immunofluorescence analysis using anti-HA and anti-Flag antibodies. **(E)** RNF5-mediated degradation of Core is independent of its E3 enzyme activity. HepG2 cells were co-transfected with HA-Core and Flag-RNF5-C42S or EV. Cell lysates were analyzed by Western blotting at 30 and 48 h. **(F)** RNF5 promotes degradation of Core lysine mutants (K7A, K96A, K7/96A). Schematic representation of Core and its lysine residues (upper panel). HepG2 cells were co-transfected with wild-type or mutant Core plasmids and RNF5 or EV, and protein levels were analyzed by immunoblotting (lower panel). **(G)** RNF5 interacts with endogenous proteasome 26S subunit non-ATPase 2 (PSMD2). Co-IP analysis confirmed this interaction in HepG2 cells co-transfected with Flag-RNF5 or EV, along with GST-Core or EV. **(H)** Effect of RNF5 on the ubiquitination of Core. 293T cells were transfected with HA-Core, Flag-ubiquitin (Flag-Ub), and Flag-RNF5 expression plasmids for 30 h, followed by collection and Co-IP analysis.

The HBV Core protein plays a crucial role in the viral life cycle. To investigate how RNF5 inhibits HBV replication, we examined its impact on Core protein stability. Co-transfection of RNF5 and Core expression plasmids into HepG2 cells resulted in a significant reduction in Core protein levels, as detected by Western blotting (Figure 4A). Co-immunoprecipitation (Co-IP) assays confirmed that Flag-tagged RNF5 interacted with the Core protein (Figure 4B), and this interaction was further validated in additional Co-IP assays using endogenous RNF5 (Figure 4C). Immunofluorescence assays also demonstrated that RNF5 colocalized with the Core protein, including the RNF5 C42S mutant (Figure 4D). To determine whether RNF5 mediates Core degradation via its E3 ubiquitin ligase activity, we co-transfected the RNF5 C42S mutant with Core. Similar to wild-type RNF5, the mutant also reduced Core protein levels, suggesting that degradation occurs independently of ubiquitination (Figures 4D,E). Moreover, mutation of lysine residues within the Core protein had no effect on RNF5-mediated degradation (Figures 4E,F). Additionally, the 26S proteasome was not significantly recruited to the Core-RNF5 complexes (Figure 4G), and we confirmed that RNF5 does not enhance the ubiquitination of the Core protein (Figure 4H). These results indicate that RNF5 mediates Core degradation via a ubiquitination-independent mechanism, which may contribute to its inhibition of HBV replication.

### 3.5 RNF5 degrades HBV core protein via the caspase-3 pathway

**FIGURE 5:**
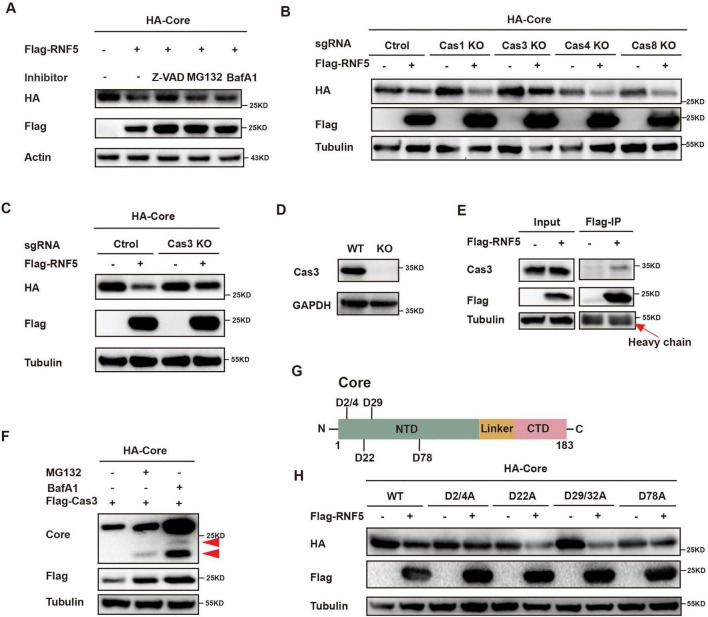
RNF5 degrades HBV core protein via the caspase-3 pathway. **(A)** Z-VAD inhibits RNF5-mediated Core degradation. HepG2 cells were co-transfected with HA-Core and Flag-RNF5 or EV, treated with Z-VAD (20 μM), MG132 (20 μM), or BafA1 (2 μM) for 6 h, and analyzed by immunoblotting. **(B,C)** Caspase-3 knockout (KO) prevents RNF5-mediated Core degradation. Caspase-1, -3, -4, and -8 were individually knocked out in HepG2 cells via CRISPR/Cas9. WT or KO cells were co-transfected with HA-Core and Flag-RNF5 or EV, and protein levels were analyzed by Western blotting. **(D)** Validation of Caspase-3 KO in HepG2 cells by immunoblotting. **(E)** RNF5 interacts with endogenous Caspase-3. Co-IP confirmed interaction in 293T cells transfected with Flag-RNF5 or EV. **(F)** 293T cells were co-transfected with HA-Core and Flag-Caspase-3 plasmids for 24 h, followed by treatment with or without MG132 (10 μM) or BafA1(1 μM) for an additional 24 h. The cells were then harvested, and whole-cell lysates were subjected to immunoblotting with the indicated antibodies. **(G)** Schematic representation of Core and its aspartate residues. **(H)** RNF5-Mediated Degradation of Core Is Inhibited by Alanine Substitutions at Aspartate Residues (D2/4A, D78A). HepG2 cells were co-transfected with wild-type or mutant Core plasmids and RNF5 or EV, and protein levels were analyzed by immunoblotting.

To further identify the pathway involved in RNF5-mediated Core degradation, we treated HepG2 cells with inhibitors of the proteasome, lysosome and caspases. The reduction of Core protein was abrogated by treatment with the pan-caspase inhibitor Z-VAD, but not by proteasome or lysosome inhibitors, suggesting the involvement of caspases (Figure 5A). Further investigation using CRISPR/Cas9 knockout of caspase-1, -3, -4, and -8 showed that only Caspase-3 knockout prevented RNF5-mediated Core degradation (Figures 5B-D). Co-IP assays confirmed that RNF5 interacts with endogenous Caspase-3 (Figure 5E). The cleaved form of the core protein by Caspase-3 was detected using a core antibody in the presence of MG-132 or BafA1, both of which inhibit the degradation of cleaved proteins (Figure 5F). These data indicate that Caspase-3 can cleave the core protein, and following cleavage, the cleaved core protein is subsequently detected by the proteasome and lysosome. Moreover, mutation analysis of Core protein revealed that three cleavage sites (D2/4 and D78) are critical for Caspase-3-mediated degradation (Figures 5G,H). These findings indicate that RNF5 facilitates Core protein degradation through a Caspase-3-dependent pathway, thereby inhibiting HBV replication.

## 4 Discussion

Our study demonstrates that RNF5 inhibits HBV replication by promoting the degradation of the Core protein through a Caspase-3-dependent mechanism. Importantly, this antiviral effect is independent of RNF5’s E3 ligase activity, suggesting an alternative mode of action. The upregulation of RNF5 in HBV patients further supports its potential role in the host antiviral response. Targeting RNF5 or enhancing its activity may provide new therapeutic strategies for the treatment of HBV infection.

The findings of this study add to the growing body of evidence supporting the significance of targeting the HBV Core protein in antiviral therapy. CAMs that target capsid assembly and promote Core protein misfolding have shown promising results in preclinical and clinical studies (McFadden and Sarafianos, 2023; Popping et al., 2019; Taverniti et al., 2024). Similarly, our findings highlight the potential of exploiting RNF5-mediated degradation of the Core protein to inhibit HBV replication. Given that RNF5 acts independently of ubiquitination and employs a Caspase-3 pathway, it may serve as a valuable adjunct or alternative to existing CAM-based therapies.

RNF5 has previously been implicated in antiviral responses against several viruses, such as SARS-CoV-2, where it was shown to degrade viral structural proteins (Kong et al., 2022; Li Z. et al., 2023; Zhong et al., 2009; Zhong et al., 2010). Further supporting the antiviral role of RNF5, Yuan et al. demonstrated that RNF5 facilitates SARS-CoV-2 membrane protein-mediated virion release, illustrating its involvement in different stages of the viral life cycle (Yuan et al., 2021). Moreover, RNF5 has been shown to regulate antiviral responses by mediating degradation of the adaptor protein MITA (mediator of IRF3 activation, also called STING, stimulator of IFN genes), which is involved in immune signaling (Zhong et al., 2009). The diverse roles of RNF5 in viral inhibition suggest that enhancing its activity could have broad-spectrum antiviral effects, making it an attractive target for therapeutic intervention. Our findings expand this role to HBV and provide a basis for future research into the application of RNF5 as a therapeutic target against HBV and potentially other viruses. Moreover, the identification of specific cleavage sites on the HBV Core protein by Caspase-3 opens new avenues for developing drugs that can selectively enhance this degradation pathway, providing an additional layer of specificity for targeting HBV-infected cells. While this study primarily utilized artificially constructed HBV to investigate the role of RNF5 in HBV replication, future studies using authentic HBV will provide additional validation of our findings and offer a more comprehensive understanding of RNF5’s role in the virus’s replication.

The involvement of Caspase-3 in RNF5-mediated Core protein degradation is intriguing, as caspases are typically associated with apoptotic processes (Collins et al., 2024; Fu et al., 2024). While there was previous study demonstrates that the Caspase-3-mediated cleavage of 14-3-3eta generates a truncated form (sub-14-3-3eta) that antagonizes melanoma differentiation-associated gene 5 (MDA5)-dependent type I interferon induction, highlighting a mechanism by which viruses impair antiviral immunity and regulate inflammatory homeostasis by the caspase pathway (Chan et al., 2024). And here in our study, our results suggest that RNF5 may interact with components of the apoptotic machinery to exert its antiviral effects, raising the possibility that RNF5 could influence both viral replication and host cell fate. Further studies are needed to determine whether the activation of Caspase-3 by RNF5 is directly linked to apoptosis or if it represents a non-apoptotic function of caspases in the context of HBV infection. Understanding these nuances will be critical for designing therapeutic strategies that maximize antiviral efficacy while minimizing potential cytotoxic effects.

In conclusion, our findings indicate that RNF5-mediated degradation of the HBV Core protein represents a novel antiviral mechanism that is independent of the canonical ubiquitin-proteasome pathway. This unique mode of action may provide a basis for the development of new therapeutic approaches that target the HBV Core protein through alternative degradation pathways. Given the challenges associated with eradicating HBV cccDNA, strategies that can effectively inhibit viral replication and reduce cccDNA levels are urgently needed. RNF5, either alone or in combination with other antiviral agents such as CAMs, may hold promise for achieving a functional cure for chronic hepatitis B.

## 5 Conclusion

This study reveals a novel mechanism by which RNF5 inhibits HBV replication through the degradation of Core protein via Caspase-3 (Graphical Abstract). These findings provide new insights into the antiviral properties of RNF5 and suggest that it may serve as a promising target for HBV therapy.

## Data Availability

The original contributions presented in the study are included in the article/Supplementary material, further inquiries can be directed to the corresponding authors.
