# Supplementary material for: RNF5 inhibits HBV replication by mediating caspase-3-dependent degradation of core protein
Source: Front Microbiol. 2025 Apr 1;16:1548061. doi: 10.3389/fmicb.2025.1548061 (PMC11996839; doi:10.3389/fmicb.2025.1548061)
Supplement: Supplementary file 1 [file Data_Sheet_1.docx]

**Supplementary Table 1. Clinical characteristics of HBV-infected patients**

|  |  |  | **HBV-infected patients** | | | | |
| --- | --- | --- | --- | --- | --- | --- | --- |
| No. | Sex | Age (years) | AST (U/L) | ALT (U/L) | HBV DNA (copies/mL) | HBsAg (IU/mL) | HBeAg (S/CO) |
| 1 | M | 70 | 130.6 | 185.3 | 55300 | >250 | 0.42 |
| 2 | F | 46 | 57 | 56.9 | 355000 | 49813.76 | 1052 |
| 3 | M | 33 | 33.1 | 76.2 | 33700 | / | / |
| 4 | M | 42 | 27 | 34.7 | 244000 | / | / |
| 5 | M | 43 | 119.7 | 116 | 83500000 | 4978.35 | 984.091 |
| 6 | M | 56 | 25.6 | 20.4 | 282 | 1342.31 | / |
| 7 | F | 39 | 54.3 | 92.2 | 3580 | 23128.46 | / |
| 8 | F | 51 | 20.8 | 16.7 | 276 | 16008.67 | / |
| 9 | M | 57 | 48 | 62.5 | 63.6 | 30.14 | / |
| 10 | F | 39 | 18.3 | 21.5 | 170000000 | 67932.55 | 1805.888 |
| 11 | M | 64 | 53.1 | 33.3 | 126 | >250 | 0.351 |
| 12 | F | 33 | 40.7 | 62.3 | 102 | 72.9 | 0.312 |
| 13 | M | 40 | 52.6 | 101.8 | 2620 | 2477.24 | 0.342 |
| 14 | F | 26 | 36 | 46.7 | 294 | 25078.67 | / |
| 15 | F | 46 | 228.6 | 347.5 | 21200000 | >250 | 1.741 |
| 16 | F | 41 | 29.2 | 32.6 | 118000 | 2065.64 | / |
| 17 | M | 57 | 252.1 | 63.6 | 8420 | 40.55 | / |
| 18 | F | 50 | 18.2 | 14.8 | 740 | >250 | 0.451 |
| 19 | F | 29 | 17.1 | 20.9 | 1380 | 343.04 | / |
| 20 | M | 22 | / | / | 121000000 | 79262.71 | / |
| 21 | F | 49 | 14.9 | 3.5 | 9560 | >250 | 0.118 |
| 22 | F | 20 | 71 | 88 | 27000000 | 12951.13 | / |
| 23 | M | 35 | 62 | 45.8 | 69.8 | 4088.35 | / |
| 24 | F | 42 | 684.3 | 534.3 | 7010000 | / | / |
| 25 | F | 50 | 34 | 32.9 | 3580 | 3117.27 | / |
| 26 | M | 42 | 91.2 | 127 | 375 | 1414.16 | / |
| 27 | F | 63 | 55 | 33.5 | 114000000 | >250 | 915.267 |
| 28 | F | 47 | 31.2 | 19.2 | 879 | 2655.45 | 0.38 |
| 29 | M | 55 | 61.1 | 54.4 | 1970 | / | / |
| 30 | M | 52 | 41.4 | 52.3 | 305000 | 8515.93 | 1.492 |
| 31 | M | 36 | 35.4 | 42.9 | 25400 | 10965.84 | / |
| 32 | M | 37 | 30.7 | 37.6 | 439000000 | 72226.72 | 1480.39 |
| 33 | M | 50 | 98.8 | 151.3 | 2180 | 342.4 | 0.451 |
| 34 | M | 51 | 42.8 | 48.7 | 1280000 | 3736.25 | 23.547 |
| 35 | F | 57 | 25.1 | 20.2 | 173000 | 8549.05 | / |
| 36 | M | 24 | 18.2 | 16.9 | 332000000 | >250 | 1567.955 |
| 37 | M | 57 | 184 | 130 | 505 | >250 | 824.993 |
| 38 | M | 34 | 59.9 | 118.8 | 119000000 | 23885.9 | / |
| 39 | F | 41 | 26.4 | 21.6 | 12000 | 11650.86 | / |
| 40 | M | 24 | 29.7 | 43 | 62700000 | 71298.23 | / |
| 41 | F | 40 | 53.1 | 53.4 | 346000 | 5775.62 | 0.418 |
| 42 | M | 51 | / | / | 1280000 | 383.13 | / |
| 43 | M | 62 | / | / | 137000 | 1453.17 | / |
| 44 | M | 38 | 75.6 | 184.1 | 140000 | 6222.78 | / |

**Supplementary Table 2. Clinical characteristics of healthy controls**

|  |  |  | **Healthy controls** | |
| --- | --- | --- | --- | --- |
| NO | Sex | Age/year | AST (U/L) | ALT (U/L) |
| 1 | M | 51 | 35.1 | 46.4 |
| 2 | F | 56 | 46.1 | 59.6 |
| 3 | M |  | 26.2 | 18.4 |
| 4 | M | 52 | 19.3 | 19.4 |
| 5 | F | 34 | // |  |
| 6 | F | 34 | / | / |
| 7 | M | 69 | / | / |
| 8 | F | 45 | / | / |
| 9 | M | 43 | / | / |
| 10 | F | 51 | / | / |
| 11 | F | 32 | / | / |
| 12 | M | 18 | / | / |
| 13 | M | 31 | / | / |
| 14 | F | 46 | / | / |
| 15 | M | 27 | / | / |
| 16 | M | 18 | / | / |
| 17 | F | 26 | / | / |
| 18 | F | 45 | / | / |
| 19 | F | 25 | / | / |

**Supplementary Table 3. Comparison of demographic and clinical characteristics between HBV-infected patients and healthy controls**

| **Indicators** | **Group** | **n** | **Mean ± SD / n (%)** | **t / χ²** | **P** |
| --- | --- | --- | --- | --- | --- |
| Sex | Male | 34 | 9 (47.3%) vs 25 (56.8%) | 0.0722 | 0.0722 |
|  | Female | 29 | 10 (52.7%) vs 19 (43.2%) |  |  |
| Age (years) | Healthy controls vs HBV | 63 | 39.26 ± 13.65 vs 44.11 ± 11.97 | 1.415 | 0.1621 |

**Supplementary Table 4. Primer sequences used for infusion cloning, sgRNAs, and qPCR**

| **Infusion Cloning Primers** | |
| --- | --- |
| GENE | PRIMER |
| Core cloning F | TCGCGGCCGCTCTAGAATGGACATTGACCCGTAT |
| Core cloning R | AGGCGCCTGGTCTAGAGCCCTTAGAGTTACAATC |
| K7A F | TCGCGGCCGCTCTAGAATGGACATTGACCCGTATGCAGAATTTGGAGC |
| K96A F | AATATGGGCCTAGCAATCAGACAACTA |
| K96A R | TAGTTGTCTGATTGCTAGGCCCATATT |
| D2/4A F | TCGCGGCCGCTCTAGAATGGCCATCGCCCCTTATAAAG |
| D22A F | TTTTTGCCTTCTGCATTCTTTCCTTCT |
| D22A R | AGAAGGAAAGAATGCAGAAGGCAAAAA |
| D29/32A F | CCTTCTATTCGAGCTCTCCTCGCCACCGCCTCTGCT |
| D29/32A R | AGCAGAGGCGGTGGCGAGGAGAGCTCGAATAGAAGG |
| D78A F | AGTAATTTGGAAGCTCCAGCATCCAGG |
| D78A R | CCTGGATGCTGGAGCTTCCAAATTACT |
|  |  |

**sgRNAs**

| GENE | Forward | Reverse |
| --- | --- | --- |
| Caspase-1 | ATGACGCCTTGCCCTCATA | TTATCTAATACATCTGGGA |
| Caspase-3 | ATGACGCCTTGCCCTCATA | TTATCTAATACATCTGGGA |
| Caspase-4 | ATGGCGACGCTGGCAAAG | TTACCTGAGGGCGAGGAA |
| Caspase-8 | CACCTCCTTTGCGGAATGTAGTCC | AAACGGACTACATTCCGCAAAGGA |

**Q-PCR primers**

| GENE | Forward | Reverse |
| --- | --- | --- |
| GAPDH | CGGATTTGGTCGTATTGGG | TCTCGCTCCTGGAAGATGG |
| HBVDNA | GAGTGTGGATTCGCACTCC | GAGGCGAGGGAGTTCTTCT |
| pgRNA | TCTTGCCTTACTTTTGGAAG | AGTTCTTCTTCTAGGGGACC |
| RNF5 | GCCAGAACGGCAAGAGTGT | GGCTCCCTCGCCCATAAAG |
